# Septum of the penis – dissection, anatomical description and functional relevance

**DOI:** 10.1186/s12610-024-00235-0

**Published:** 2024-11-12

**Authors:** Florin-Mihail Filipoiu, Radu-Tudor Ion, Zoran-Florin Filipoiu, Adrian-Daniel Tulin, Octavian Enciu, Mihaly Enyedi

**Affiliations:** 1 Morphological Sciences Department, Anatomy Discipline, University of Medicine and Pharmacy “Carol Davila” Bucharest, Bucharest, Romania; 2 Doctoral School of the University of Medicine and Pharmacy “Carol Davila” Bucharest, Eroii Sanitari Blvd, no. 8, Bucharest, 050474 Romania; 3 Surgery Department, University of Medicine and Pharmacy “Carol Davila” Bucharest, Bucharest, Romania

**Keywords:** Pectiniform septum of the penis, Dissection, Corpus cavernosum, Corpus spongiosum, Septum glandis, Distal ligament, Septum pectiniforme du Pénis, Dissection, Corps caverneux, Corps spongieux, Septum glandis, Ligament distal

## Abstract

**Background:**

The septum of the penis or the pectiniform septum (from Latina pecten) is a connective structure that separates the two corpora cavernosa of the penis. It is formed through the joining of the circular fibers of the tunica albuginea, which envelops the corpora cavernosa. The septum neither completely separates, nor entirely joins the two corpora cavernosa.

**Results:**

We dissected the penile septum in 10 formalized bodies. The dissections were carried out using magnifying lenses, emphasizing the connective structures. We studied the structure of the septum using transverse and sagittal dissection planes. We identified the penile septum as a structure consisting of clusters of tendinous cords incompletely separating the two cavernous structures. The septum completely separates the two corpora cavernosa in its posterior segment. As we progress forward, the septum starts resembling the tendinous cords that attach to the papillary muscles of the heart. These cords are differentiated from the internal layer of the albuginea of each corpus cavernosum. We evaluated the opportunity of considering the anterior and posterior intercavernous ligaments as septal structures.

**Conclusion:**

In this type of construction, the septum maintains both the hemodynamic and mechanical coherence of the cavernous structures and allows penile movement more efficiently than a continuous septal structure. The septum enables the lengthening of the penis and simultaneous filling with blood of both its corpora cavernosa through the transseptal vascular anastomosis. This allows for penile deformation during erection to be avoided. Our study also provides a description of the way the corpora cavernosa attach to the bulbus of corpus spongiosum.

## Résumé

**Contexte:**

Le septum du pénis, ou septum pectiniforme (du latin *pecten*), est une structure conjonctive qui sépare les deux corps caverneux du pénis. Il est formé par la jonction des fibres circulaires de la tunique albuginée, qui enveloppe les corps caverneux. Le septum ne sépare pas complètement, ni ne relie entièrement, les deux corps caverneux.

**Résultats:**

Nous avons disséqué le septum pénien de 10 cadavres qui avaient déjà fait l’objet d’un traitement au formol. Les dissections ont été réalisées à l’aide de loupes, en mettant l’accent sur les structures conjonctives. Nous avons étudié la structure du septum à l’aide de plans de dissection transverses et sagittales. Nous avons identifié le septum pénien comme une structure composée de grappes de cordons tendineux séparant incomplètement les deux structures caverneuses. Le septum sépare complètement les deux corps caverneux dans son segment postérieur. Au fur et à mesure que nous progressons, le septum commence à ressembler aux cordons tendineux qui s’attachent aux muscles papillaires du cœur. Ces cordons sont dissociés de la couche interne de l’albuginée de chaque corps caverneux. Nous avons estimé l’opportunité de considérer les ligaments intercaverneux antérieurs et postérieurs comme des structures septales.

**Conclusion:**

Dans ce type de construction, le septum maintient à la fois la cohérence hémodynamique et mécanique des structures caverneuses, et il permet le mouvement du pénis plus efficacement qu’une structure septale continue. Le septum permet l’allongement du pénis et le remplissage simultané de sang de ses deux corps caverneux par l’anastomose vasculaire transseptale. Cela permet d’éviter la déformation du pénis pendant l’érection. Notre étude fournit également une description de la façon dont les corps caverneux se fixent au bulbe du corps spongieux.

## Introduction

Regarding the penile structure, nature had to solve the following biological problem: on one hand, how can a tubular organ allow free flow of the sperm, increasing its volume and hardness during erection, and on the other hand, how can the same organ be flaccid and small in size at sexual rest, as well as during urination.

The problem is extremely difficult and, in principle, presupposes the existence of a functional hemodynamic complex associated with an integrative mechanical structure to bring together the cavernous formations. This structure is represented by the penile septum, known as the pectiniform septum (Latin: comb) or pectiniform or penile septum, or as part of the penile connective system [1].

The septum is the result of the structural differentiation of the penis and is represented by the joining of the circular fibers of the tunica albuginea surrounding ​​the two corpora cavernosa [2, 3]. The bilateral character of the disposition of the circular fibers remains obvious throughout the entire length of the septum. Regarding septal anatomy and function, little information is presented in the literature. It is known that the septum separates the corpora cavernosa and is denser posteriorly. The explicit information regarding septal function is that the septum is a separating structure.

There are numerous questions that have not been asked. Why does the septum look like a comb, how accurate is this comparison, what is the actual involvement of the septum in penile biomechanics. In our opinion, the anatomy and importance of the penile septum deserves re-evaluation.

The classic literature recognizes the pectiniform septum as the only septal structure in the penis. However, there are other separating structures that could be considered belonging to the septum. Anteriorly there is the glandular septum and posteriorly there is a mass of dense connective tissue that separates the roots of the corpora cavernosa [4]. We will present each of these structures in turn.

## Material and methods

In the laboratory of the Anatomy Department at the 'Carol Davila' University of Medicine and Pharmacy, dissections were conducted on ten cadavers. The cadavers had previously undergone formalization by injection into the femoral artery with a 10% formalin solution, followed by a 30-day preservation in tanks containing the same formalin concentration [5].

The dissections were carried out in anatomical layers, during which the pectiniform septum was identified. Its anatomical distribution and behavior were then described. Photographs of the dissection pieces were taken, and subsequent discussions ensued. The images were digitally edited without altering the scientific content.

## Results and discussion

**Fig. 1:**
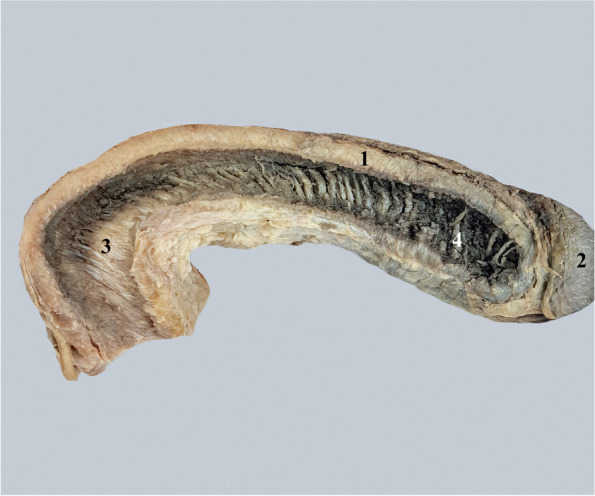
Lateral view of the pectiniform septum. 1. Albuginea of the right cavernous body. 2. Glans penis. 3. Posterior part of the pectiniform septum. 4. Anterior part of the pectiniform septum

The existence of three cavernous structures in the anatomy of the penis creates the need for both their separation as well as their functional reunification. The integrative structure that accomplishes this is the penile septum itself. It is formed by joining the circular fibers of the tunica albuginea of the two corpora cavernosa. The septum does not have the appearance of a complete wall, but on the contrary, it has a fenestrated appearance [4, 6, 7] Fig. 1.

**Fig. 2:**
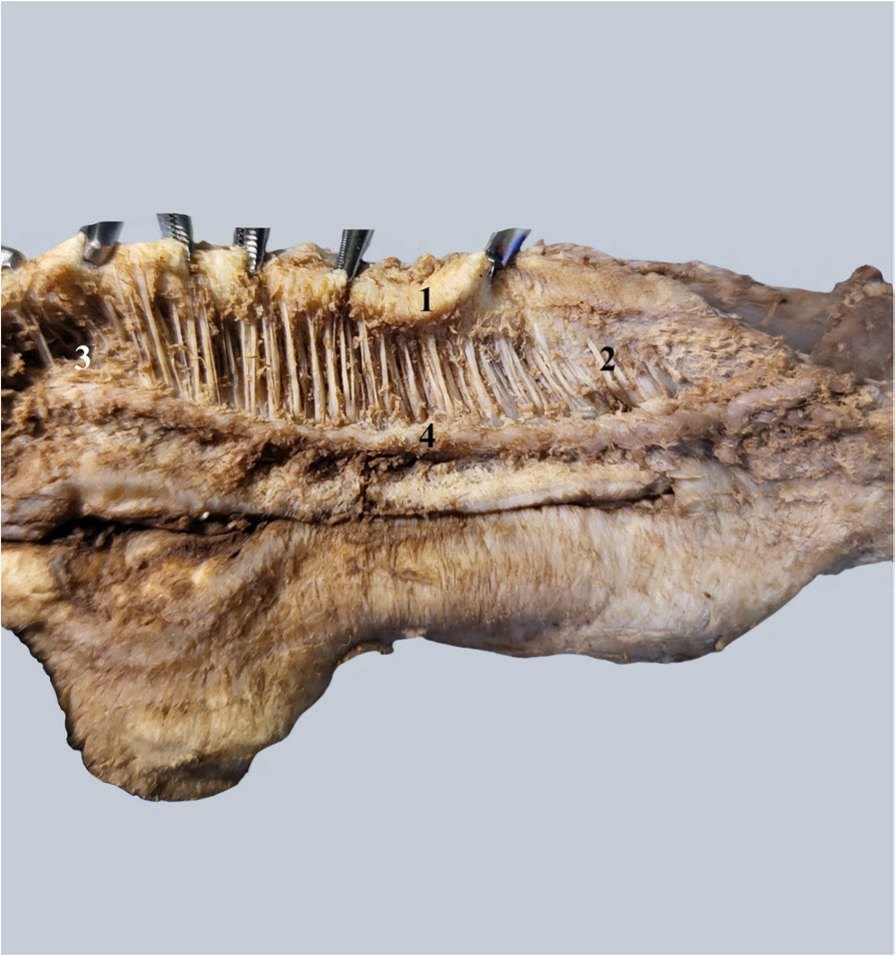
Detail of the pectiniform septum. 1. Albuginea of the left corpus cavernosum. 2. Posterior part of the pectiniform septum. 3. Anterior part of the pectiniform septum with rarer tendinous cords. 4. Cavernous artery

In the posterior part, the fenestrations are rarer, and the septum is denser. Structurally, the septum is made up of pairs of chordae tendineae, similar to the chordae tendineae in the heart Fig. 2.

**Fig. 3:**
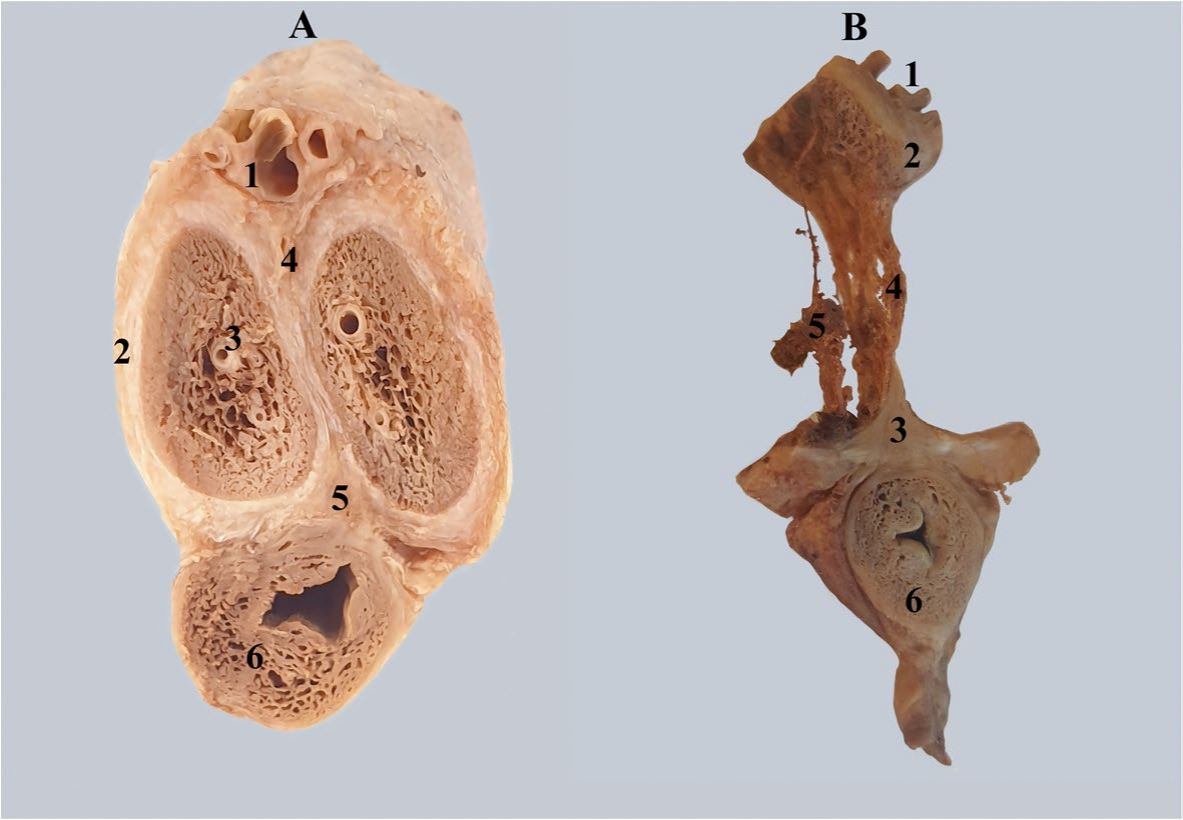
A. Transverse cross-section through the penile body. 1. Dorsal vascular-nervous bundle. 2. Albuginea of the left corpus cavernosum. 3. Cavernous artery. 4. Superior triangular connective mass joining the two corpora cavernosa. 4. Corpus spongiosum and urethra. B. Demonstration of the two triangular masses after removal of the corpora cavernosa. 1. Dorsal vascular-nervous bundle. 2. Connective mass joining superiorly the two corpora cavernosa. 3. Tendinous center of the penis. 4. Tendinous cords of the pectiniform septum. 5. Cavernous artery and its branches. 6. Corpus spongiosum and urethra

They are placed superiorly and inferiorly on two dense connective masses that occupy the triangular space formed by joining the albuginea of ​​the corpora cavernosa [2, 3, 8, 9] Fig. 3.

**Fig. 4:**
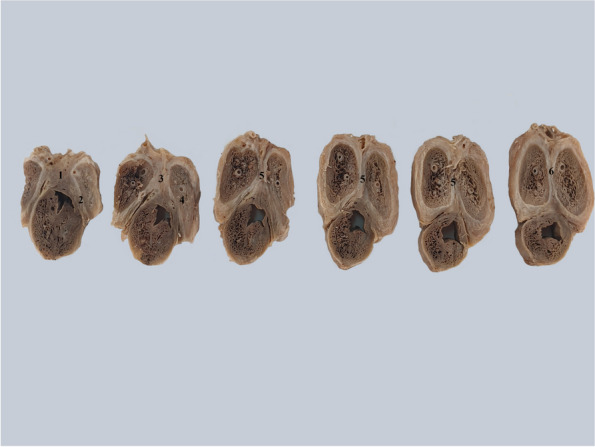
Transverse cross-sections through the penile body from posterior to anterior. 1. Posterior intercavernous ligament. 2. Root of the left corpus cavernosum. 3. Posterior part of the pectiniform septum. 4. Right corpus cavernosum. 5. Pectiniform septum. 6. Pectiniform septum at the anterior extremity of the corpora cavernosa

This is particularly visible in cross-sections through the penile body Fig. 4.

**Fig. 5:**
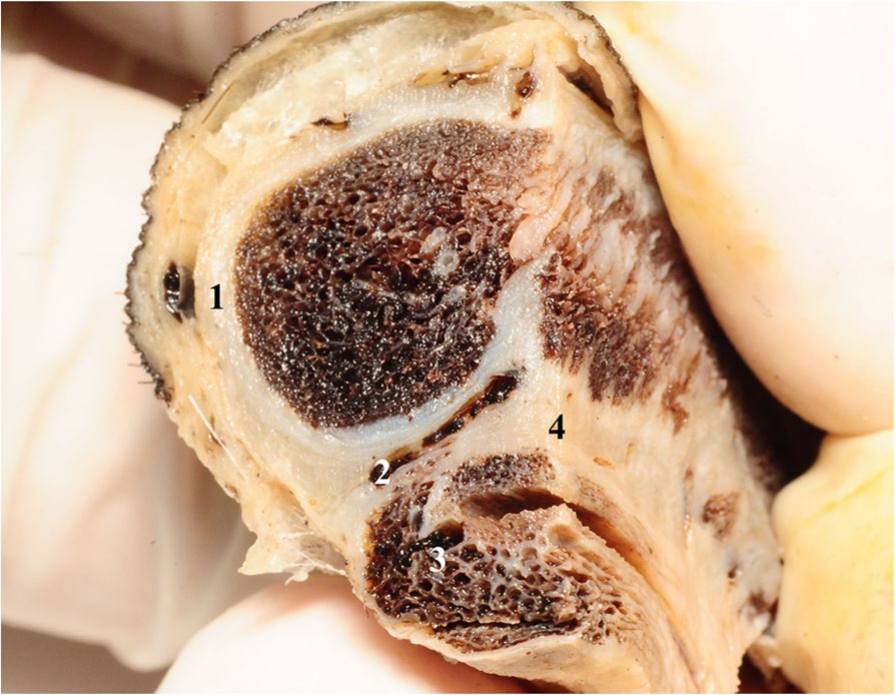
Sagittal cross-section through the penile body to highlight the tendinous center of the penis. 1. Albuginea of the left corpus cavernosum. 2. Veins between the corpus spongiosum and the corpora cavernosa. 3. Corpus spongiosum and urethra. 4. Tendinous center of the penis

The best represented is the connective mass that joins the two corpora cavernosa at the base of the septum, but at the same time, reunites the corpora cavernosa with the dorsal surface of the corpus spongiosum Fig. 5.

**Fig. 6:**
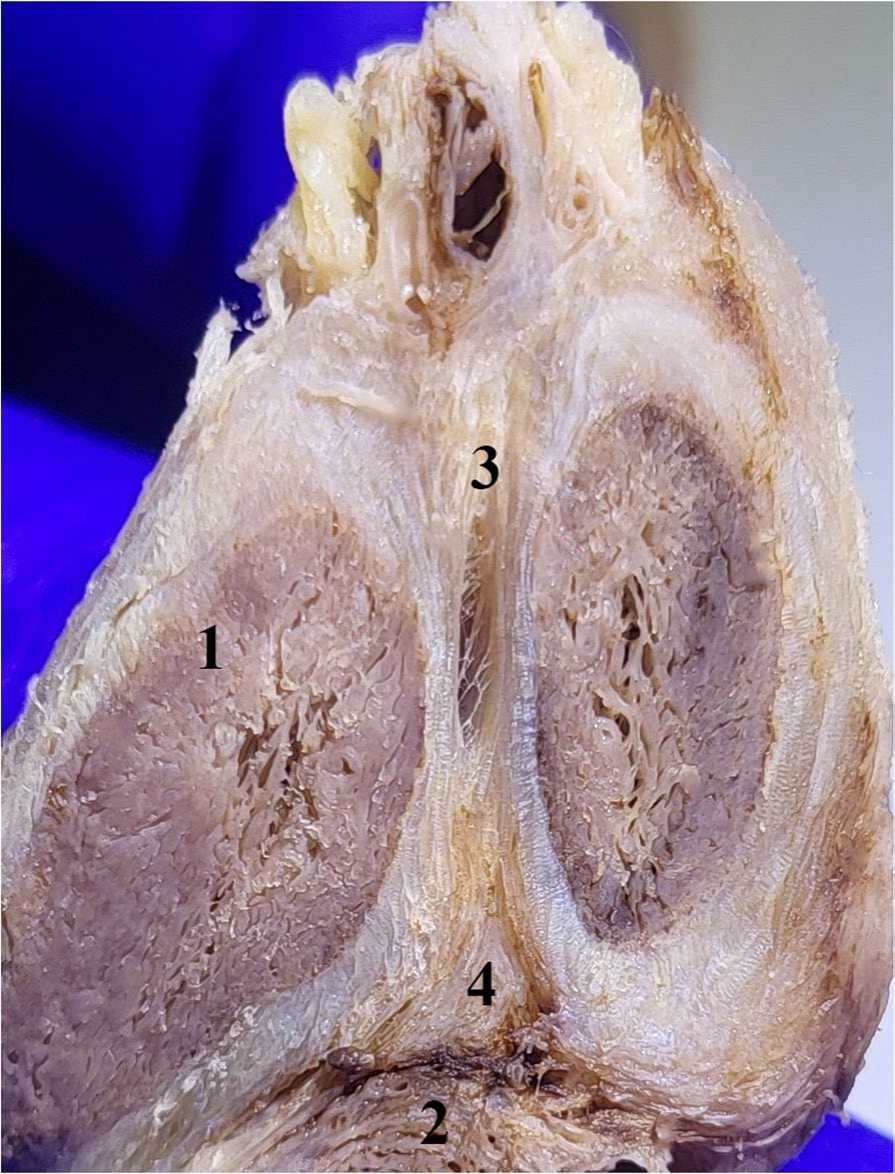
Transverse cross-section through the penile body to highlight the tendinous center. 1. Left corpus cavernosum. 2. Corpus spongiosum. 3. Superior triangular connective mass. 4. Tendinous center that joins the corpora cavernosa and the corpus spongiosum

This connective structure, triangular in cross-section, is actually the main mechanical integrative structure for the three cavernous structures [10]. If we make a comparison with the terminology used in the anatomy of the perineum, it is a kind of tendinous center of the penis where all the important connective structures reach. Dr. Hsu refers to it as a “ventral thickening” of the albuginea, but we consider the term “tendinous center” more appropriate due to its importance in keeping together all the cavernous structures of the penis during erection [10] Fig. 6.

**Fig. 7:**
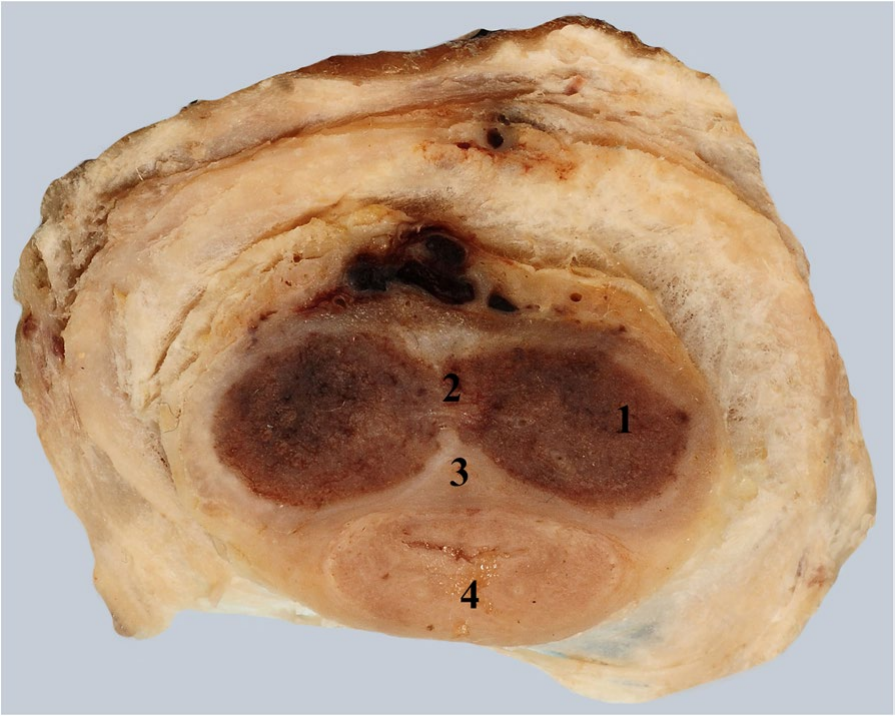
Transverse cross-section that highlights the trans-cavernous anastomosis. 1. Right corpora cavernosa. 2. Intercavernous transverse veins at the level of the pectiniform septum. 3. Tendinous center of the penis. 4. Corpus spongiosum and urethra

This connective mass extends along the entire length of the penis between the corpora cavernosa and the dorsal surface of the corpus spongiosum. It is crossed by venous structures [1]. The dense connective mass in the upper part of the penile septum is not as well represented as the lower connective mass. Through the spaces between the septal tendinous cords, the cavernous tissue makes right-left anastomoses [1, 6] Fig. 7.

**Fig. 8:**
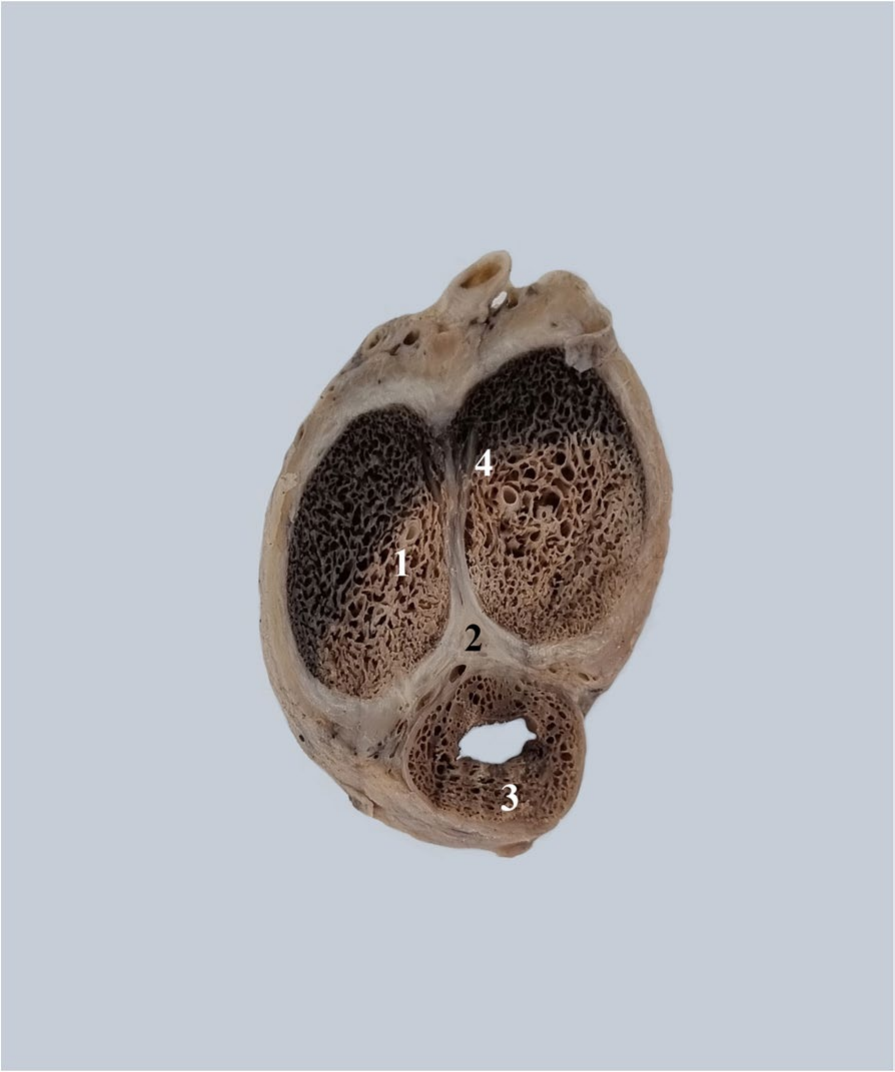
1. Left corpus cavernosum and the cavernous artery. 2. Tendinous center. 3. Corpus spongiosum and urethra. 4. Vertically directed veins in the pectiniform septum

Sometimes these transseptal anastomoses are made through venous structures. These anastomoses explain the fact that, in dynamics, the two corpora cavernosa can vary in length simultaneously. In the width of the septum, among the tendinous cords, we identified small veins with a vertical arrangement [11] Fig. 8.

**Fig. 9:**
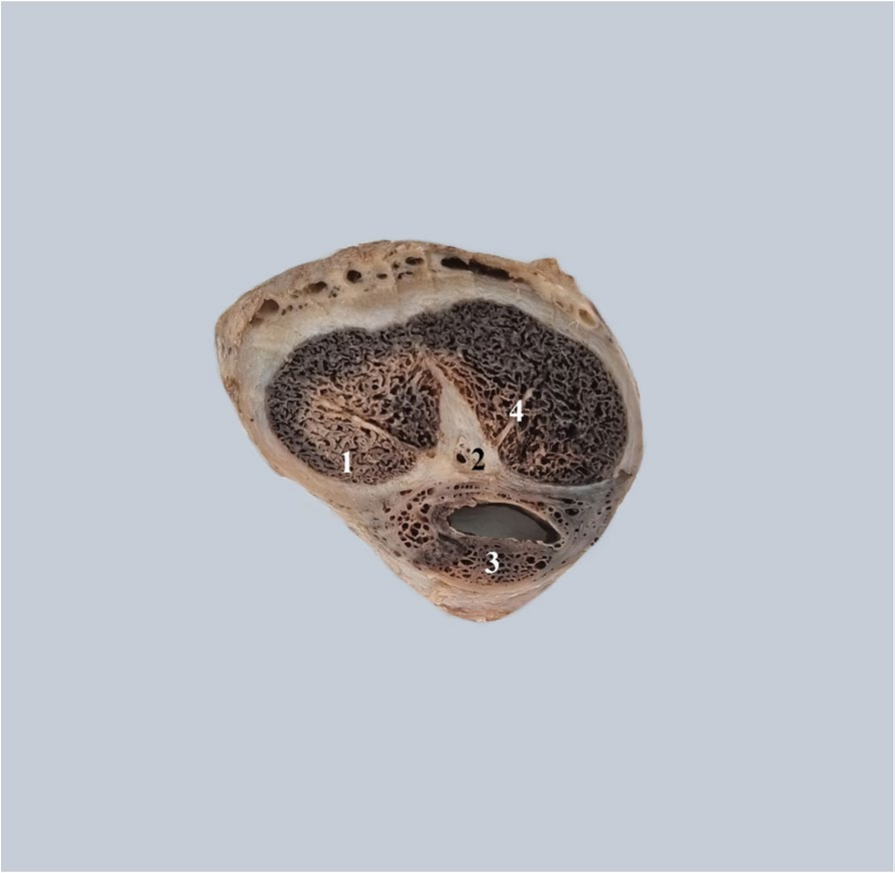
1. Left corpus cavernosum. 2. Tendinous center with veins. 3. Corpus spongiosum and urethra. 4. Pillars

From place to place, from the base of the penile septum, tendinous cords that appear to insert on the internal face of the albuginea depart obliquely towards the dorsal surface of the corpora cavernosa. In appearance, these cords seem to strengthen the penile connective complex. In reality, these tendon pillars are too small to hold the albuginea in tension [4, 7–9, 12] Fig. 9.

**Fig. 10:**
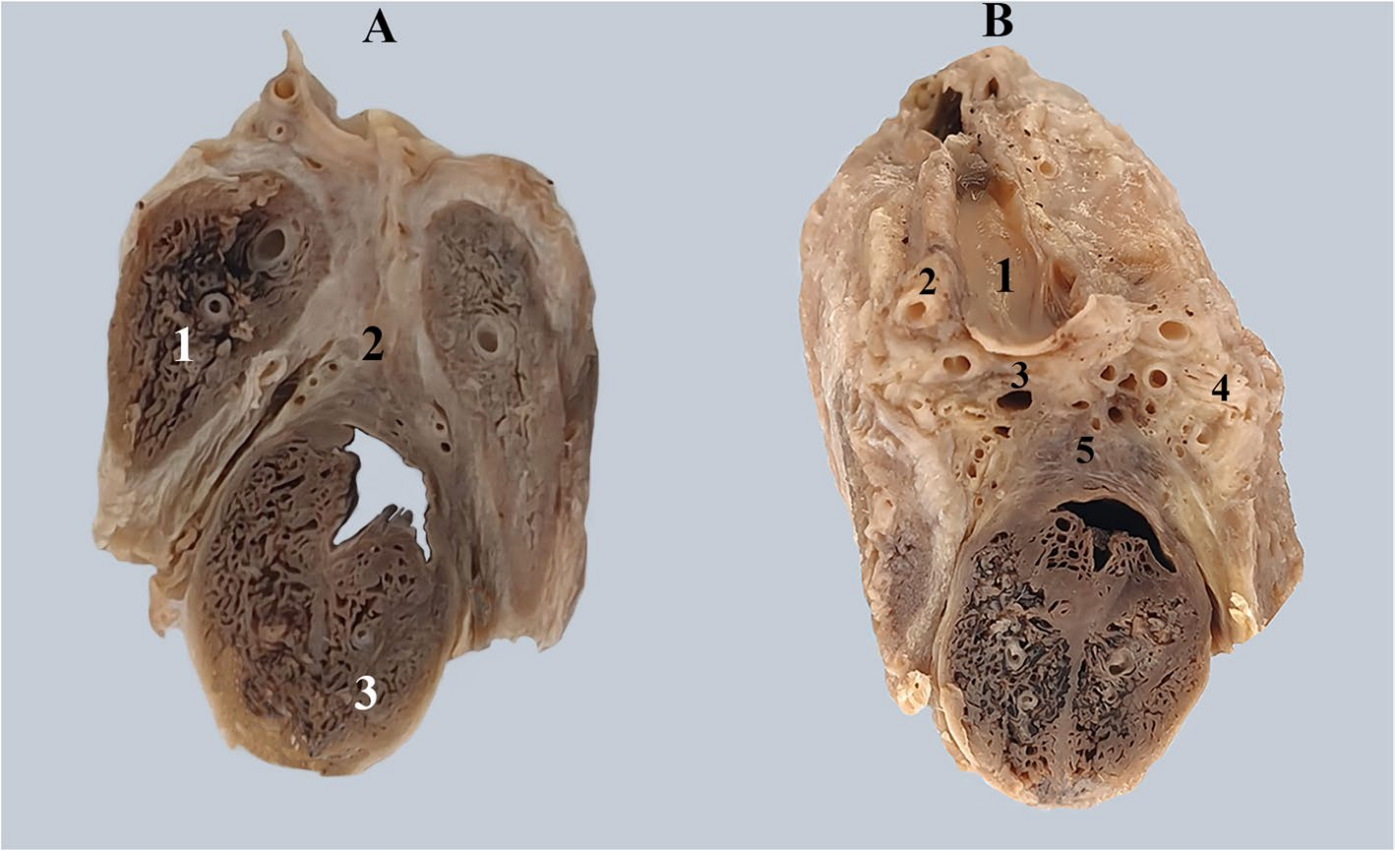
Section demonstrating the space between the roots of the corpora cavernosa containing the posterior intercavernous ligament and penile vessels. A. 1. Left corpus cavernosum. 2. Posterior intercavernous ligament. 3. Corpus spongiosum and urethra. B. 1. Sectioned superficial dorsal vein. 2. Dorsal artery. 3. Deep dorsal veins. 4. Dorsal nerve. 5. Posterior intercavernous ligament

At the level of the penis, however, there are other median structures with a right-left separating function. Although there is an impulse to classify them as part of the penile septum, we believe that these structures should be described separately from the pectiniform septum. Thus, we describe:

A) Posteriorly, where the roots of the corpora cavernosa diverge, there is a mass of dense connective tissue with a right-left separating function. This connective mass has had various names over time. In old anatomy books, such as "Traité d'Anatomie humaine' by L. Testut" we find it under the name of the posterior intercavernous transverse ligament [13] Fig. 10.

This “ligament” attaches the bulb of the penis to the corpora cavernosa and is basically a continuation of the tendinous center between the roots of the two corpora cavernosa. Other authors call this connective mass the "hilum of the penis" because it is traversed by the cavernous vessels on their way to the corpora cavernosa [6, 14, 15]. However, this connective mass may be very well considered as a posterior segment of the penile septum in its entirety.

**Fig. 11:**
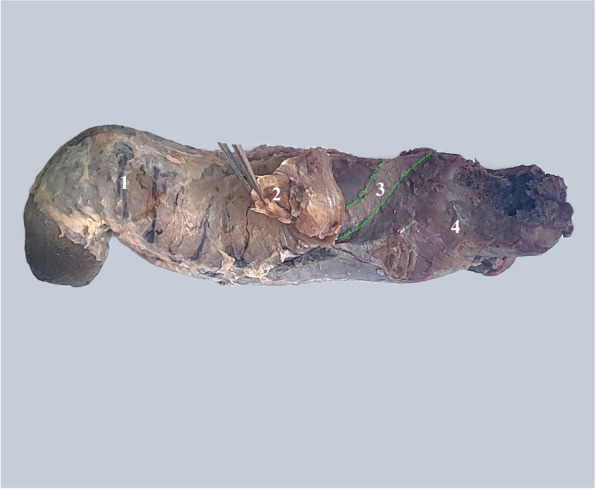
1. Body of the penis with circumferential veins. 2. The root of the left corpus cavernosum grasped in tweezers and reflected after sectioning its insertion on the corpus spongiosum. 3. Attachment of the root of the corpus cavernosum on the corpus spongiosum (between the green dotted lines). 4. Bulbus spongiosum covered by the bulbospongiosus muscle

In the context of the description of the local anatomy, we draw attention to how the roots of the cavernous bodies are fixed to each other by the intercavernous ligament and then adhere to the lateral faces of the anterior end of the spongy bulb [13] Fig. 11.

Basically, the corpora cavernosa, the roots of the corpora cavernosa and the bulbus spongiosum at the base of the penis are united by the transverse intercavernous septum, and in its continuation, by fibrous insertions of the roots of the corpora cavernosa.

B) At the level of the gland, the glandular septum has been previously described by some authors [16]. The descriptions, however, are not convincing enough. In the “Traité d'Anatomie humaine” by L. Testut a structure of dense connective tissue called the transverse intercavernous ligament (anterior) is described in the glans penis, superior to the urethra and between the anterior extremities of the corpora cavernosa [13].

**Fig. 12:**
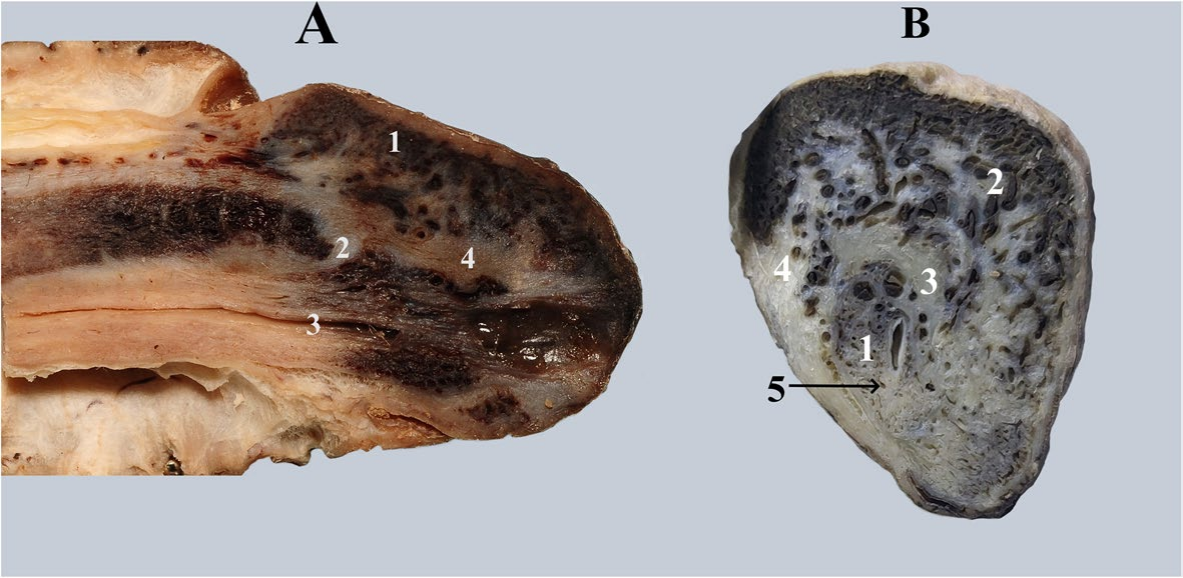
**A **Sagittal cross-section through the glans of the penis. 1. Caverns inside the glans. 2. Apex of the corpus cavernosum and its albuginea. 3. Urethra. 4. Conjunctive fascicles in continuation of the albuginea of the corpus cavernosum, which extend almost to the extremity of the glans. **B **Transverse cross-section through the glans of the penis, distal to the apex of corpora cavernosa. 1. Urethra surrounded by the caverns of corpus spongiosum. 2. Caverns inside the glans. 3. Connective mass with predominantly transverse direction. 4. Connective fascicles in the continuation of the albuginea of the corpora cavernosa. 5. Septum glandis

In the same region, Geng-Long Hsu describes a variable sagittal septum on the midline above the glandular urethra. He also hypothesizes that these structures originate from the longitudinal fibers of the albuginea of ​​the corpora cavernosa [4]. According to our observations, none of the opinions are completely true to reality. We identified connective masses in the thickness of the supra-urethral gland with variable appearances, dispositions and sizes. We cannot consider these connective structures as septal Fig. 12.

"On the ventral aspect of the urethra, Dr.Özbey describes a septal structure called the septum glandis, which is a median structure within the glans, extending between the ventral aspect of the urethra, the frenulum, and the tunica albuginea. This structure, which is to be considered during hypospadias reconstruction surgery, can be observed in Fig. 12.B [17–19].

In the morpho-functional evaluation of the penis, the penile septum has so far been regarded as a structure with a simple mechanical role. In reality, the septum and its components have an integrative role in penile anatomy and erectile function. Due to the fenestrated aspect, it allows for right-left vascular communication, making possible the dynamic coherence of the cavernous structures during erection (that is, the dimensions of the two corpora cavernosa change simultaneously). The fenestrated aspect gives the pectiniform septum capabilities to modify penile length that would not be present if the septum were a compact structure. If the septum were compact, without fenestrations, the penis would not be able to elongate sufficiently during erection. The base of the pectinate septum runs through the body of the penis as a real tendinous center that joins the corpus spongiosum to the two corpora cavernosa. In this way, the urethra follows the length changes of the cavernous structures.

The existence of connective structures with a potential right-left separation located at the extremities of the pectinate septum has been observed in anatomical studies since the early 1900s [13]. Over time, they have also been observed by other authors and we also present them on dissection pieces. However, their integration into a structural theory was not proposed [4]. We refer to the posterior intercavernous ligament/penile hilum and the anterior intercavernous ligament/septum of the glans.

We believe that these connective formations represent support zones that provide support to the extremities of the pectinate septum. At the level of the glans, the term “anterior intercavernous ligament” is a blanket term for the connective tissue masses at this level and we do not recommend using the term “glandular septum”. For the connective tissue between the albuginea and the dorsal aspect of the glandular urethra. However, there is a septum glandis ventral to the urethra.

At the base of the septum, the term "hilum of the penis" is more metaphorical, since most of the vessels and nerves of the penis remain superficial and do not enter the corpora cavernosa. The term "posterior intercavernous ligament" seems more appropriate, but is insufficiently correlated with its function. The posterior intercavernous ligament is part of the ligamentous system of the roots of the corpora cavernosa. This ligament solidifies the roots of the corpora cavernosa and fixes them to the upper part of the bulbus spongiosum. The roots of the corpora cavernosa attach into the continuation of the ligament, on the lateral faces of the bulbus of the penis.

We specify that we have not found in the specialized literature any reference regarding the insertion method of the root of the corpora cavernosa on the bulbus spongiosum. This notion has so far been missed from the description of the anatomical logic of the penis. Basically, it was missing from the description of the way the roots of the corpora cavernosa attach on the bulbus spongiosum.

## Conclusions

In conclusion, we want to emphasize the role of the pectiniform septum in maintaining the symmetry of the penis in dynamics and in maintaining penile straightness in erection. The ability of the septum to change dimensions during erection and the hemodynamics of the penile cavernous system explain the ability of the penis to change its dimensions. This behavior would not be possible if the septum was a compact structure, resulting from the fusion of the albuginea of ​​the two corpora cavernosa.

## Data Availability

The cadavers used in this study were provided by the Anatomy Discipline of the Morphology Department of the University of Medicine and Pharmacy “carol Davila” Bucharest.

## Data availability

No datasets were generated or analysed during the current study.
